# The development and validation of the Ethical Sensitivity Questionnaire for Nursing Students

**DOI:** 10.1186/s12909-019-1625-8

**Published:** 2019-06-17

**Authors:** Taeko Muramatsu, Mieko Nakamura, Eisaku Okada, Harumi Katayama, Toshiyuki Ojima

**Affiliations:** 1 Department of Fundamental Nursing, Hamamatsu University School of Medicine, 1-20-1 Handayama, Higashi-ku, Hamamatsu city, Shizuoka, 4313192 Japan; 2 Department of Community Health and Preventive Medicine, Hamamatsu University School of Medicine, 1-20-1 Handayama, Higashi-ku, Hamamatsu city, Shizuoka, 4313192 Japan

**Keywords:** Ethics education, Ethical sensitivity, Questionnaire development, Student nurse

## Abstract

**Background:**

Recent advances in medicine and an increasingly demanding healthcare environment are causing various complicated ethical problems. Nursing students need to prepare to deal with ethical issues in their future roles. Ethical sensitivity is a key aspect of the ethical decision-making process; however, there is no scale to measure nursing students’ ethical sensitivity. Therefore, we developed a scale and verified its reliability and validity.

**Methods:**

The Ethical Sensitivity Questionnaire for Nursing Students (ESQ-NS) was developed in three phases. First, questionnaire items were formulated after a literature review and interviews with nursing students. Next, its face and content validity were examined by an expert panel and piloted among nursing university graduates. Then, a final draft questionnaire survey was administered to nursing university students from 10 Japanese universities in 2015 and an exploratory factor analysis was performed. Criteria-related relevance was examined to compare established scales (i.e. the Japanese version of the Moral Sensitivity Test (JMST) and the Japanese version of the revised Moral Sensitivity Questionnaire (JMSQ)) using single regression analysis. A second questionnaire survey was conducted in one of the 10 universities to examine reliability.

**Results:**

Initially, 48 items including ethical conflict in clinical nursing practice were formulated, and 47 items were approved by the expert panel. Five-hundred and twenty-eight nursing students responded to the final draft questionnaire. Participants’ mean age was 20.4 (standard deviation = 3.1) years. The questionnaire was reduced to 13 items and three factor structures were determined by exploratory factor analysis: ‘respect for individuals’, ‘distributive justice’, and ‘maintaining patients’ confidentiality’. The Cronbach’s alpha values for items in each domain ranged from 0.77–0.81, and the Cronbach’s alpha for the entire ESQ-NS was 0.82. The ESQ-NS was significantly associated with specific domains: ‛Judgment of the care conflict’ from the JMST and ‘Sense of Moral Burden’ from the JMSQ. Pearson’s correlation coefficient of the ESQ-NS between the first and second survey was 0.42 (*p* < .01).

**Conclusions:**

The EAQ-NS, which was developed to evaluate the ethical susceptibility of nursing students, showed good validity, internal consistency, and reliability. This questionnaire can be used to evaluate nursing students’ ethics education by self-evaluation.

## Background

Recent advances in medicine and an increasingly demanding healthcare environment are causing various complicated ethical problems. Therefore, medical professionals are required to have a high ethical capability, and the importance of medical ethics in health professional education is increasing [1, 2]. Fry and Johnstone [3] acknowledged the importance of ethical practice in providing quality care, and described ethical competence as one of the professional components. Nurses are expected to not only play the role of a specialist opinion coordinator for smooth cooperation of the medicine team by bridging patient care, but also that of an advocate for patients between doctors and patients. Since the health care profession has a distinct culture, including its values [4], nurses who are coordinating the medicine team are frequently confronted with conflicting values, especially ethical problems [5–15].

For that reason, as healthcare professionals, nurses are expected to display a prominent level of practical ethical skills and respect the values and rights of patients who need their professional care [16, 17]. To solve ethical problems, nurses first require ethical sensitivity, which is the ability to recognize ethical problems. Rest [18] defined ethical sensitivity as a precursor to making ethical decisions. Those who display ethical sensitivity can assess the responses and feelings of others and are aware of potential courses of action. Lützén and colleagues [19] defined moral sensitivity as the ability to recognize a moral conflict, show a contextual and intuitive understanding of the patient’s vulnerable situation, and have insight into the ethical consequences of the decisions made.

Previous research on ethics in nursing practice strongly indicated the need to prepare nursing students to meet ethical challenges in their future role as nurses. For that reason, nursing students require ethical sensitivity training during nursing education. Therefore, most nursing schools in Japan have incorporated mandatory courses on medical ethics [20]. These course objectives typically include increasing students’ understanding of ethical norms and resolving ethical dilemmas in clinical settings. However, today, there is no established scale to evaluate nursing ethics education in Japan.

To measure the students’ ethical ability, various methods, such as subjective reporting and standardized examination including clinical vignettes, were developed [21–23]. As a concept that is still developing, different authors have used distinct terms such as moral sensitivity and ethical sensitivity [18, 19, 24, 25]; however, these have been noted as interchangeable [25]. Weaver et al. [26] defined ethical sensitivity as ‘the capacity to decide with intelligence and compassion, given uncertainty in a care situation, drawing as needed on a critical understanding of codes for ethical conduct, clinical experience, academic learning and self-knowledge, with an additional ability to anticipate consequences and the courage to act’ (p. 610). In this research, instead of ‘subjective judgment’ based on student’s personal feelings and preferences, we emphasized ‘objectivity’ based on the situation and the setting. Therefore, we defined the concept of ethical sensitivity of nursing students as ‘the ability to recognize ethical issues in nursing practice’. Several measures have been developed to assess the ethical sensitivity of nurses and healthcare workers [19, 27, 28]. However, most of these scales were developed for nurses or professionals, and therefore, to our knowledge, there is no published scale to measure the ethical sensitivity of nursing students. Therefore, we developed a scale to measure nursing students’ ethical sensitivity and examined its reliability and validity.

## Methods

### Development of the Ethical Sensitivity Questionnaire for Nursing Students (ESQ-NS)

The initial pool of questionnaire items was created through an extensive review of literature on source of ethical conflict and ethical issue in nursing, as well as interviews with 4th year nursing students [29]. In this review, the databases used included the Cumulative Index to Nursing and Allied Health Literature (CINAHL), Google Scholar, CiNii Articles, and Japan Medical Abstracts. Keywords included ‘nursing students’ or ‘student nurse’, ‘ethical issues’, ‘moral sensitivity’, ‘ethical sensitivity’, and ‘nursing practice’. Initial searches included articles published between 2000 and 2014. Reports were excluded if they did not focus on ethical issues or ethical sensitivity, and if they did not focus on nursing students. Furthermore, unpublished articles were also excluded. We arbitrarily selected some literature from papers found through the use of search terms, and examined it. At the time of the search, we focused on ethical problems that students could encounter in clinical nursing practice. For that reason, we excluded the problems associated with life-sustaining treatment, including maintenance of life-sustaining devices and ethical issues related to bioethics such as prenatal diagnosis. Consequently, we confirmed 48 items including ethical conflicts. These items consisted of informed consent, confidentiality, patients’ interests, professional values, and patients’ privacy. All items included ethical issues encountered by Japanese nursing students in clinical practice.

Face and content validity of the 48 items were examined by an expert panel, which consisted of five academicians: two university nursing ethics faculty members, one clinical nursing faculty member, and two fundamental nursing faculty members. After verification by the expert panel, five nursing university graduates without clinical experience reviewed the semantic contents of the items. Forty-five of the 48 items were evaluated as ‘easy to understand’; however, two items were noted as ‘difficult to understand’. Therefore, we revised the wording of these two items. In addition, one item was deleted for redundancy; finally, the expert panel approved 47 questionnaire items.

Responses to each questionnaire item were recorded on a 4-point Likert type scale: ‘Do you feel each of the following items poses an ethical problem? Please circle the appropriate response’ (1 = I do not think at all, 2 = I do not think much, 3 = I think a little, 4 = I think very). Scores ranged from 47 to 188 points and higher scores indicated greater ethical sensitivity.

### Ethical sensitivity by established scales

Ethical sensitivity by established scales was measured using the Japanese version of the Moral Sensitivity Test (JMST) and the Japanese version of the revised Moral Sensitivity Questionnaire (JMSQ). These scales measure the ethical sensitivity of nurses and were originally developed by Lützén and colleagues [19, 27, 28], and adapted to be culturally relevant to Japanese nurses by Nakamura and colleagues [30] and Maeda and colleagues [31]. The JMST is composed of seven subdomains (responsibility of nurses and with respect of patient, faithfulness to judgment of doctor and rule, introspection, sincerity, judgment of the care conflict, decision-making, and benevolence). The JMSQ is composed of three subdomains (moral strength, sense of moral burden, and moral responsibility).

### Sample and data collection

Questionnaire surveys were administered in 10 universities, which were willing to participate in the survey, among 29 nursing universities in the Chubu district belonging to the Japanese nursing-related university council. Questionnaires (*N* = 2480) were distributed to students through staff and teachers at each university. The cover letter included information about the procedure and right to refusal. Return of these papers indicated students’ consent to participate. The survey was mailed to the nine universities, and was conducted using the retention method in one university. Test-retest reliability for instrument stability was conducted 8 weeks following the original testing on 55 participants in one university. The survey was conducted from April to September 2015. The Hamamatsu University School of Medicine’s Institutional Review Board approved this study (E14–347).

### Demographic data

Demographic measures included age, sex, school year, ethical education, and ethical knowledge.

### Statistical analyses

Initially, descriptive statistics were analysed. Second, an exploratory factor analysis (EFA) [32] was conducted to find possible factor structures of questionnaire items after checking response bias of items by ceiling and floor effects. For internal consistency reliability, Cronbach’s alpha [33] was calculated for each factor. For criterion-related validity, a simple liner regression analysis was performed using the total and individual scores of the domains of JMST and JMSQ as independent variables, and the total and individual scores of the domains of ESQ-NS as dependent variables. Test-retest reliability was analysed using Pearson’s correlation analysis [34]. The average of the total and individual scores for the domains of ESQ-NS were compared by grade using a one-way analysis of variance. IBM Statistical Package for the Social Sciences (SPSS) Statistics 22 was used to calculate descriptive statistics and to conduct the EFA.

### Sample size

Sample size was calculated based on a rule of thumb, considering 10 participants per item in the ESQ-NS to perform the factor analysis [31]. Therefore, the number of participants required was 47*10 = 470.

### Reliability

Cronbach’s alpha was used to assess the internal consistency of the items in the ESQ-NS (for all items and each domain). Cronbach’s alpha values greater than 0.9 suggest redundancy of some items, values between 0.70–0.90 imply adequate internal consistency, values between 0.50–0.69 indicate poor internal consistency, and values below 0.50 indicate unacceptable internal consistency [33].

Test-retest reliability was assessed using Pearson’s correlation coefficient. Pearson’s r-values can range from − 1 to + 1. The closer the absolute value is to 1, the more linearly it is related. When it is close to 1, there is a positive linear relation; when it is close to − 1, there is a negative linear relation. Pearson’s correlation coefficient values can be interpreted as follows: |r|: 0–0.20 = little, if any correlation; |r|: 0.21–0.40 = low correlation; |r|: 0.41–0.70 = moderate correlation; and |r|: 0.71–1 = high correlation [34].

## Results

### Respondents’ characteristics

Five-hundred and twenty-eight questionnaires were returned, out of which 525 were valid (three were excluded for missing values). Participants were mostly women (95.2%). All school years were included (Table 1). The mean value of the JMSQ was 4.2 (SD = 0.44) and the mean value of the JMST was 3.9 (SD = 0.32). Missing data from 47 items were substituted with the average values of individual answers.

**Table 1:** Demographic characteristics of participants (*n* = 525)

Characteristics	N	%
Gender		
Female	500	95.2
Male	22	4.2
Unanswered	3	0.6
Age	mean 20.4 (SD 3.1) years	
School Year		
1st grade	144	27.4
2nd grade	141	26.9
3rd grade	83	15.8
4th grade	152	29.0
Unanswered	5	0.9
Types of universities		
National and public	364	69.3
Private university	161	30.7

### ESQ-NS

Before the EFA, the 47 items were refined by checking distribution of the responses. Items were excluded if: (i) mean score plus one standard deviation was larger than 4, or mean score minus one standard deviation was smaller than 1; and (ii) there were correlation coefficients of 0.8 or more in the item correlation matrix. Accordingly, 25 items were eliminated; scores of 23 items were 3.4 or more, and correlation coefficients of two pairs were 0.8 or more. The resulting 22 items were retained. Since all items set as reversal items were excluded through this process, the remaining 22 items were all positive question items.

Next, the EFA with maximum-likelihood factor analysis and promax rotation was conducted. Three criteria were used in selecting the number of factors: (i) a scree-Test: a scree plot that showed a distinct change between the steep slope of the large factors and a gradual trailing of the remaining factors; (ii) a Cronbach’s alpha of 0.70 or more; and (iii) a possibility of factor interpretation. Two criteria were used in selecting the number of items within a factor (i) an item-factor loading of 0.4 or more; and (ii) all items with higher loading on one factor. Consequently, 9 of the 22 items were eliminated, leaving 13 items that loaded on the three factors (Table 2).

**Table 2:** Exploratory factor analysis (n = 525)

		Factor		
		1	2	3
Factor 1: Respect for individuals (Cronbach’s α = 0.81)				
1	Railing is placed around a bed to prevent the patient from falling out.	**0.68**	–0.1	–0.04
2	Although a postoperative patient has refused postural changes due to pain, postural changes are performed to prevent postoperative complications.	**0.63**	0.10	–0.02
3	Although a terminally ill patient has refused postural changes due to respiratory discomfort caused by moving, postural changes are performed every two hours due to the high risk of pressure ulcers.	**0.60**	0.10	–0.01
4	An elderly patient who had said he/she wanted to go home was placed in a facility because he/she had no relatives who could care for them at home.	**0.59**	–0.04	0.00
5	A sensor mat is placed at the bedside of a patient who had fallen once in the ward.	**0.56**	0.19	–0.05
6	You allowed a patient with dementia to stay at the nurses’ station while sitting in a wheelchair with the safety-belt fastened.	**0.55**	–0.06	0.06
7	A patient under your care who was of the opposite sex had refused to let you watch over him/her when he/she showered; however, you did so after persuading him/her to allow you to.	**0.45**	–0.09	0.12
8	To administer medication to a patient with dementia who refuses medication, it is mixed with a drink without the patient’s knowledge.	**0.41**	0.01	0.10
Factor 2: Distributive justice (Cronbach’s α = 0.79)				
9	A terminally ill patient wished to use the bathroom for elimination; therefore, two nurses took the patient to the bathroom and aided.	–0.13	**0.96**	–0.01
10	A bedridden patient who had always received a bed bath pleaded to take a regular bath; therefore, three nurses assisted the patient in taking a regular bath.	–0.03	**0.76**	0.02
11	To accommodate the eating speed of patients with dysphagia, you provide eating assistance that involves uninterrupted supervision for at least one hour.	–0.10	**0.65**	0.11
Factor 3: Maintaining patients’ confidentiality (Cronbach’s α = 0.77)				
12	Reporting the condition of a patient under your care to the nurse in charge in a multi-bed hospital room.	–0.02	–0.04	**0.83**
13	Reporting the details of the patient care to a clinical leader in the corridor.	0.05	0.04	**0.75**
	Factor contribution	3.51	2.78	1.59
	Correlations among factors			
	1	1	0.57	0.28
	2		1	0.16
	3			1

Factor loading > 0.4 are a bold face

Factor 1 consisted of eight items in conjunction with the ethical conflict that related to the respect for autonomy and non-maleficence and beneficence. This factor included items such as ‘you allowed a patient with dementia to stay at the nurses’ station while sitting in a wheelchair with the safety belt fastened’ and ‘to administer medication to a patient with dementia who refuses medication, it is mixed with a drink without the patient’s knowledge’. Therefore, we named factor 1 ‘respect for individuals’.

Factor 2 consisted of three items in conjunction with the ethical principle that related to justice and beneficence and non-maleficence. This factor included items such as ‘a bedridden patient who had always received a bed bath strongly wished to take a regular bath; therefore, three nurses assisted the patient in taking a regular bath’ and ‘to accommodate the eating speed of patients with dysphagia, you provide eating assistance that involves uninterrupted supervision for at least one hour’. Therefore, we named factor 2 ‘distributive justice’.

Factor 3 also consisted of two items in conjunction with the obligation of confidentiality. This factor included items such as ‘reporting the details of the patient care to a clinical leader in the corridor’ and ‘reporting the condition of a patient under your care to the nurse in charge in a multi-bed hospital room’. Therefore, we named factor 3 ‘maintaining patients’ confidentiality’. Correlations among factors ranged between 0.16–0.57 and Cronbach’s alphas ranged between 0.77–0.81. Cronbach’s alpha of overall ESQ-NS was 0.82.

### Reliability and validity of ESQ-NS

The results of the test-retest revealed that Pearson’s correlation coefficient of the overall ESQ-NS was 0.42, Factor 1 (‘respect for individuals’) was 0.41, Factor 2 (‘distributive justice’) was 0.27, and Factor 3 (‘maintaining patients’ confidentiality) was 0.34 (Table 3).

**Table 3:** Verification of reliability of the ESQ-NS using a test-retest method (n=55)

Pearson’s correlation coefficient	
Overall	0.42**
Factor 1: Respect for individuals	0.41**
Factor 2: Distributive justice	0.27
Factor 3: Maintaining patients’ confidentiality	0.34*

* *P* <0.05

** *P <*0.01

### Criteria-related relevance

‘Respect for individuals’ and ‘maintaining patients’ confidentiality’ were significantly associated with ‘Judgment of the care conflict’, which is a subdomain of the JMST, and ‘Sense of Moral Burden’, which is a subdomain of the JMSQ. On the other hand, an inverse association was observed between ‘distributive justice’ and ‘moral responsibility’, which are subdomains of the JMSQ (Table 4).

**Table 4:** Verification of criterion-related validity of the ESQ-NS

	ESQ-NS											
	Overall			Factor 1			Factor 2			Factor 3		
				Respect for individuals			Distributive justice			Maintaining patients’ confidentiality		
	*β* ^a^	95% CI	*P*	*β* ^a^	95% CI	*P*	*β* ^a^	95% CI	*P*	*β* ^a^	95% CI	*P*
JMST												
Overall	0.078	(–0.073–0.229)	0.31	0.144	(–0.074–0.362)	0.20	0.066	(–0.360–0.492)	0.76	0.01	(–0.649–0.669)	0.98
Responsibility of nurses and with respect of patient	0.019	(–0.033–0.071)	0.48	0.060	(–0.015–0.135)	0.12	–0.138	(–0.285–0.009)	0.07	0.143	(–0.084–0.371)	0.22
Faithfulness to judgment of a doctor and rule	-0.031	(–0.081–0.020)	0.23	-0.067	(–0.140–0.005)	0.07	0.09	(–0.052–0.231)	0.21	–0.185	(–0.404–0.033)	0.10
Introspection	0.025	(–0.006–0.063)	0.11	0.055	(0.005–0.104)	0.03*	0.033	(–0.065–0.130)	0.51	–0.038	(–0.188–0.112)	0.62
Sincerity	0.025	(–0.014–0.064)	0.20	0.035	(–0.021–0.091)	0.22	0.074	(–0.036–0.183)	0.19	–0.019	(–0.188–0.151)	0.83
Judgment of the care conflict	0.049	(0.019–0.080)	<0.01**	0.078	(0.035 –0.122)	<0.01***	0.011	(–0.075–0.097)	0.81	0.197	(0.065–0.329)	<0.01**
Decision-making	0.005	(–0.024–0.035)	0.73	0.018	(–0.025–0.061)	0.40	–0.019	(–0.103–0.065)	0.66	–0.021	(–0.151–0.108)	0.75
Benevolence	–0.006	(–0.039–0.027)	0.72	–0.011	(–0.059–0.036)	0.65	0.037	(–0.055–0.130)	0.43	–0.103	(–0.246–0.040)	0.16
JMSQ												
Overall	0.034	(–0.021–0.088)	0.22	0.054	(–0.025–0.133)	0.18	0.008	(–0.145–0.162)	0.92	0.131	(–0.106–0.368)	0.28
Moral Strength	–0.010	(–0.041–0.021)	0.52	–0.011	(–0.055–0.034)	0.64	0.007	(–0.080–0.094)	0.87	–0.114	(-0.249–0.020)	0.10
Sense of Moral Burden	0.042	(0.011 –0.073)	0.01*	0.051	(0.006–0.097)	0.03*	0.055	(–0.033–0.144)	0.22	0.198	(0.062–0.335)	<0.01**
Moral Responsibility	0.002	(–0.013–0.017)	0.79	0.013	(–0.009–0.035)	0.24	–0.054	(–0.097–0.011)	0.01*	0.047	(–0.019–0.114)	0.16

*CI* confidence interval

^a^Single regression analysis

* *P* < 0.05

** *P* < 0.01

*** *P* < 0.001

### School year and ESQ-NS scores

There was a significant difference in the mean ESQ-NS overall score, Factor 1, and Factor 2 by school year. Means of overall score, Factor 1, and Factor 3 were significantly higher in 2nd, 3rd and 4th year students as compared to 1st year students. No significant difference between Factor 2 and school year were observed (Table 5).

**Table 5:** School year and ESQ-NS scores

	ESQ-NS											
	Overall			Factor 1			Factor 2			Factor 3		
				Respect for individuals			Distributive justice			Maintaining patients’ confidentiality		
	n	mean	SD	n	mean	SD	n	mean	SD	n	mean	SD
School Year												
1st grade	144	2.4	0.5a*b*c*	144	2.5	0.5a*b*c*	144	2.0	0.7	144	3.0	0.9a*b*c*
2nd grade	141	2.6	0.4a*	141	2.7	0.5a*d*	141	2.2	0.7	141	3.2	0.7a*
3rd grade	83	2.8	0.5b*	83	2.9	0.5b*d*	83	2.1	0.8	83	3.3	0.7b*
4th grade	152	2.7	0.5c*	152	2.8	0.5c*	152	2.0	0.7	152	3.3	0.6c*

One-way ANOVA

Games-Howell's multiple comparison test was applied to reveal differences

A significant difference was recognized between the same symbols (abcd)

*P<0.05

## Discussion

The ESQ-NS was developed to measure the ethical sensitivity of nursing students. It consisted of 13 items, and an EFA revealed that the ESQ-NS had 3 domains: ‘respect for individuals’, ‘distributive justice’, and ‘maintaining patients’ confidentiality’. The ESQ-NS showed good internal consistency and reasonable reliability. Furthermore, the ESQ-NS was significantly associated with school year; nursing students from upper grades showed higher ethical sensitivity than first-year students. Baykara and colleagues [35] argued that nursing students have a certain level of ethical sensitivity to begin with, but that ethical sensitivity can be further developed thorough nursing education. The ESQ-NS can possibly detect this educational achievement of ethical sensitivity among nursing students.

In addition, total ESQ-NS score was positively associated with ‘Judgment of the care conflict’ from the JMST and ‘Sense of Moral Burden’ from the JMSQ. These associations were conceptually reasonable; therefore, the ESQ-NS succeeded at showing sufficient criterion-related validity. However, total ESQ-NS score was neither significantly associated with the total JMST score nor with the total JMSQ score. Koutoku [36] reported that the JMST reflects the nurses’ subjective aspects and measures their autonomy as professionals. These factors may correspond to ‘ethical judgment’ and ‘awareness sense’ among the elements of ‘ethical sensitivity’, as indicated by Lützén [19, 27]. These results indicate that the ESQ-NS partly reflects ethical sensitivity but does not reflect ‘judgment’ and ‘awareness’. In other words, the ESQ-NS is a specific scale that measures ‘judgment of the care conflict’ and ‘sense of moral burden’.

Contrary to the original expectation, an inverse association was observed between ‘distributive justice’ and ‘moral responsibility’ in the JMSQ. The two items comprising ‘moral responsibility’ indicated, primarily, a moral obligation to work according to rules and regulations, and the insights of its purpose [30]. Maeda [31] noted that the possibility of misaligning the direction of responses regarding ‘moral responsibility’ depends on interpretation of the question; one possible interpretation is ‘they are responsible for doing the best care for nurses regardless of insufficient resources’, and the other interpretation is, ‘I am sorry I cannot provide my best care for patients due to insufficient resources’.

On the other hand, the second domain of the ESQ-NS contained items related to fair resource allocation. This includes ethical issues about not complying with rules and hospital practices to provide patients’ desired level of care. Generally, a nurse is responsible for the care of multiple patients in clinical settings; however, a nursing student is typically only responsible for the care of one patient during nursing practice in educational settings. Therefore, it is difficult for nursing students to imagine the ethical problem of fair distribution of human resources. Furthermore, nursing students tend to recognize that providing nursing care desired by patients without fair distribution of resources according to rules is ‘not an ethical problem’. Therefore, such misunderstanding about fair distribution by nursing students may result in an inverse association between ‘distributive justice’ and ‘moral responsibility’.

‘Distributive justice’ was not associated with school year. This also relates to students’ care for one patient as described above. A previous study [37] revealed that healthcare workers experience stress and ethical dilemmas because of practicing in healthcare settings that lack resources, including a severe staff shortage. Lindh et al. [38], mentioned that one nursing student group ‘permitted themselves to be emotionally touched by seeing how a nursing supervisor had to bear injustices and a heavy workload’ (p137). However, the stress felt by ‘seeing’ the nursing supervisor may not be a direct stress for the nursing students. Since nursing students may not experience these workforce stresses directly, it seems that there was no difference among school years in this factor.

The ESQ-NS is a self-administered questionnaire to measure awareness of ethical problems in nursing students’ clinical practice, which includes 13 questionnaire items with 3 domains. The ESQ-NS is a compact and easy-to-use scale. Moreover, it may encourage awareness of ethical issues through a self-assessment process. In addition, nursing teachers may evaluate ethical sensitivity achievement through education, especially given the grade difference in ‘respect for individuals’ and ‘maintaining patients’ confidentiality’. The participants in this study were similar to those in previous studies concerning ethical sensitivity. The mean total score of the MSQ of participants in this study was slightly higher than that of a Korean study [39], and slightly lower than that of an American [17] and Swedish study [40]. Using existing scales, we were unable to grasp the improvement in ethical sensitivity per grade [39, 40]; however, we could evaluate ethical sensitivity due to academic achievement using this new scale.

The sample size (*N* = 525) was considered appropriate for factor analysis. A common rule of thumb for the sample size in a factor analysis is to use 5 to 10 times the number of variables [32, 41]. The final draft of the ESQ-NS comprises 47 items, and the current data are appropriate data for exploring reliability and validity by conducting an EFA.

This study had some limitations. First, we could not ascertain an accurate response rate. We mailed 2480 questionnaires to students across 10 universities; however, since the number distributed to students at each university was unknown, it was impossible to obtain an accurate response rate. Second, although we developed a new scale to measure the ethical sensitivity of nursing students and showed its credibility and validity among Japanese nursing students, further research is needed to confirm the usefulness of ESQ-NS in other countries. The ESQ-NS may contribute to research on assessing nursing students’ ethical awareness, nursing ethics education, and the evaluation of nursing practice.

## Conclusions

The ESQ-NS is the first measurement tool to evaluate the ethical sensitivity of nursing students. We confirmed that the tool has good validity, good internal consistency, and moderate reliability. This questionnaire can be used to evaluate nursing students’ ethics education by self-evaluation. Further research is needed to refine the scale to increase the generalization of the current results.

## Data Availability

The datasets used and/or analysed during the current study are available from the corresponding author on reasonable request.

## Abbreviations

ESQ-NS: Ethical Sensitivity Questionnaire for Nursing Students
JMSQ: Japanese version of the revised Moral Sensitivity Questionnaire
JMST: Japanese version of the Moral Sensitivity Test
MSQ: Moral Sensitivity Questionnair
